# Clinical, demographic, and pharmacological predictors of medication adherence to disease-modifying therapies in multiple sclerosis: a multidimensional analysis of merged survey and claims data

**DOI:** 10.1007/s00210-026-05448-w

**Published:** 2026-05-26

**Authors:** Ahkash Thavarajasingam, Lara Marleen Fricke, Christian Krauth

**Affiliations:** 1 Hannover Medical School, Institute for Epidemiology, Social Medicine and Health Systems Research, Hannover, Germany; 2 Center for Health Economics Research Hannover (CHERH), Hannover, Germany

**Keywords:** Multiple sclerosis, Disease-modifying therapies, Medication adherence, Medication possession ratio

## Abstract

Sustained adherence to disease-modifying therapies (DMTs) is essential for achieving therapeutic benefit in multiple sclerosis (MS). Despite the availability of effective agents, adherence has been reported as variable and often suboptimal in German cohorts, and the relative influence of clinical, demographic, and pharmacological factors remains insufficiently characterised. This study therefore examined predictors of objective adherence to DMTs in people with MS and the association between subjective and objective adherence. We analysed 795 people with MS (PwMS) receiving oral, subcutaneous, or intramuscular DMTs between October 2020 and September 2021. Objective adherence was measured using the Medication Possession Ratio (MPR), and subjective adherence using a visual analogue scale. Permutation-based multivariable analyses (PERMANOVA) were used to examine associations between predefined predictors and MPR. Objective adherence averaged 88.4% (SD 18.0). Subjective adherence was substantially higher, averaging 95.9% (SD 11.6; median 100%), with a pronounced ceiling effect. Therapy-related characteristics were the only consistent predictors of adherence. Oral agents and intramuscular interferon beta-1a achieved comparable adherence (*p* = 0.90), both exceeding that of subcutaneous therapies (both *p* < 0.001); within interferons, intramuscular exceeded subcutaneous administration (*p* = 0.002). Across active substances, glatiramer acetate showed the lowest adherence and teriflunomide, sphingosine-1-phosphate (S1P) receptor modulators, and dimethyl fumarate the highest (all *p* < 0.001 vs. glatiramer acetate). Demographic, including socioeconomic, and clinical characteristics, comprising age, sex, income, education, employment, MS subtype, disease duration, and disease activity, showed no measurable association with adherence. Subjective and objective adherence correlated only weakly (Spearman’s ρ = 0.10; *p* = 0.005). Adherence patterns in MS were largely shaped by pharmacological and administration-related factors rather than by patient demographics or clinical profile. The weak correlation between subjective and objective measures supports complementing patient report with objective adherence indicators in routine care.

**Supplementary Information:**

The online version contains supplementary material available at 10.1007/s00210-026-05448-w.

## Introduction

Multiple sclerosis (MS) is a chronic immune-mediated disease affecting approximately 2.8 million people worldwide and around 280,000 in Germany (Multiple Sclerosis International Federation 2020; Titcomb et al. 2023; Bittner et al. 2025) It typically begins in early adulthood and can lead to progressive disability (Loma and Heyman 2011). Disease-modifying therapies (DMTs) reduce relapses and delay progression, and sustained treatment intake is required to achieve treatment goals such as No Evidence of Disease Activity (NEDA) (Newsome et al. 2023). DMTs vary in route of administration, dosing, and tolerability, and newer oral or high-efficacy agents generally outperform first-generation (Finkelsztejn 2014; Pardo and Jones 2017).

Adherence to DMTs remains highly variable, ranging from 27%–93% internationally, with long-term adherence in Germany estimated at only 30–40% (Hansen et al. 2015; Ben-Zacharia et al. 2023). In one German cohort only 39.9% achieved a medication possession ratio (MPR) ≥ 0.8 at 24 months, with persistence of 32.3% (Hansen et al. 2015). As neither dose–response relationships nor class-specific adherence thresholds have been established for MS, the ≥ 0.8 cut-off represents a pragmatic convention rather than an outcome-validated threshold (Baumgartner et al. 2018). Adherence of ≥ 80% is nevertheless associated with fewer relapses, reduced hospitalisation, and lower healthcare expenditure (Tan et al. 2011; Sauri-Suárez et al. 2020; Nicholas et al. 2020).

Demographic and clinical variables show inconsistent associations and limited predictive value (Washington and Langdon 2022). Behavioural and care-related factors, including medication beliefs, treatment satisfaction, and continuity of care, have been identified as independent predictors and may exceed clinical characteristics in explanatory strength (Gromisch et al. 2020; Neter et al. 2024). Treatment-related factors, such as route of administration and tolerability, also show inconsistent associations, partly reflecting confounding by indication and treatment selection (Mardan et al. 2021; D’Amico et al. 2022). Determinants of adherence are rarely examined within a unified analytic framework. Even in Germany, where universal insurance largely removes access-related confounding (Döring and Paul 2010), multidomain analyses remain scarce (Ben-Zacharia et al. 2023), and the relative contribution of clinical, behavioural, and treatment-related factors remains unclear.

Adherence to oral DMTs in real-world settings remains variable and often suboptimal, challenging the assumption that route-related convenience translates into improved adherence (Nicholas et al. 2020). Reported adherence rates depend on the method of measurement. Self-reported and pharmacy-based measures capture different aspects of medication-taking behaviour, such as perceived adherence and medication availability (Stirratt et al. 2015; McKay et al. 2017).

These clinical, demographic and socioeconomic, and pharmacological domains have rarely been examined jointly in a German cohort. This study evaluates their relative contributions to objective adherence in routine MS care and quantifies the association between subjective and objective measures. Treatment-delivery characteristics were expected to show the strongest independent associations with objective adherence, given their structural alignment with the MPR metric.

## Methods

### Study design and data sources

Analyses were conducted within the Multiple Sclerosis Patient-Oriented Care in Lower Saxony (MS-PoV) study, a multicentre project linking statutory health insurance data with patient-reported information on MS care in Lower Saxony, a federal state in north-western Germany (Krüger et al. 2021). Outpatient and inpatient diagnoses, pharmacy dispensing records, and healthcare utilisation were merged with survey data using pseudonymised identifiers. Participation required electronically obtained informed consent before accessing the online survey.

The survey was developed through a literature review and focus group discussions with people with MS (PwMS) and was pretested in individuals with and without MS. Detailed methodological descriptions are available elsewhere (Krüger et al. 2021; Fricke et al. 2024).

The study was registered in the German Clinical Trials Register (DRKS00021741). Ethical approval was granted by the Ethics Committee of Hannover Medical School (9173_BO_K_2020) and the University of Oldenburg (2020–108).

### Conceptual framework and variable classification

Variable selection was guided by Bronfenbrenner’s ecological model of health behaviour, which conceptualises adherence as the result of interacting influences at the individual, social and healthcare-system levels (Bronfenbrenner 1977). To ensure conceptual consistency with this framework, variables were operationalised into three analytic domains: clinical, demographic, and pharmacological.

To reflect interactions between pharmacological properties and route-specific administration characteristics, a composite therapy variable combining active substance and route of administration was created. Clinical and pharmacological variables, including MS disease course, comorbidities, route of administration, and active substance, are described in the main results. Full variable definitions, coding rules, and mappings (including ATC classifications) are provided in Online Resource 1 (Table 1), and the list of all DMTs is presented in Online Resource 1 (Table 2).

### Study population and eligibility criteria

All insured persons of AOK Lower Saxony, the largest statutory health insurance provider in the federal state of Lower Saxony, were eligible if they were ≥ 18 years, lived in Lower Saxony, and had a confirmed MS diagnosis. MS was identified by the presence of at least one outpatient diagnosis, supplemented by either a second outpatient diagnosis in a different quarter, an accompanying DMT prescription, or one inpatient diagnosis during 2019–2020.

For inclusion in the present analyses, individuals had to have received at least two prescriptions for an oral, subcutaneous or intramuscular DMT between October 2020 and September 2021. Intravenous therapies were excluded because their fixed infusion schedules generate no patient-initiated dispensing intervals; pharmacy-based adherence metrics such as the Medication Possession Ratio (MPR) are therefore not comparable across administration routes (Moran et al. 2019).

**Fig. 1:**
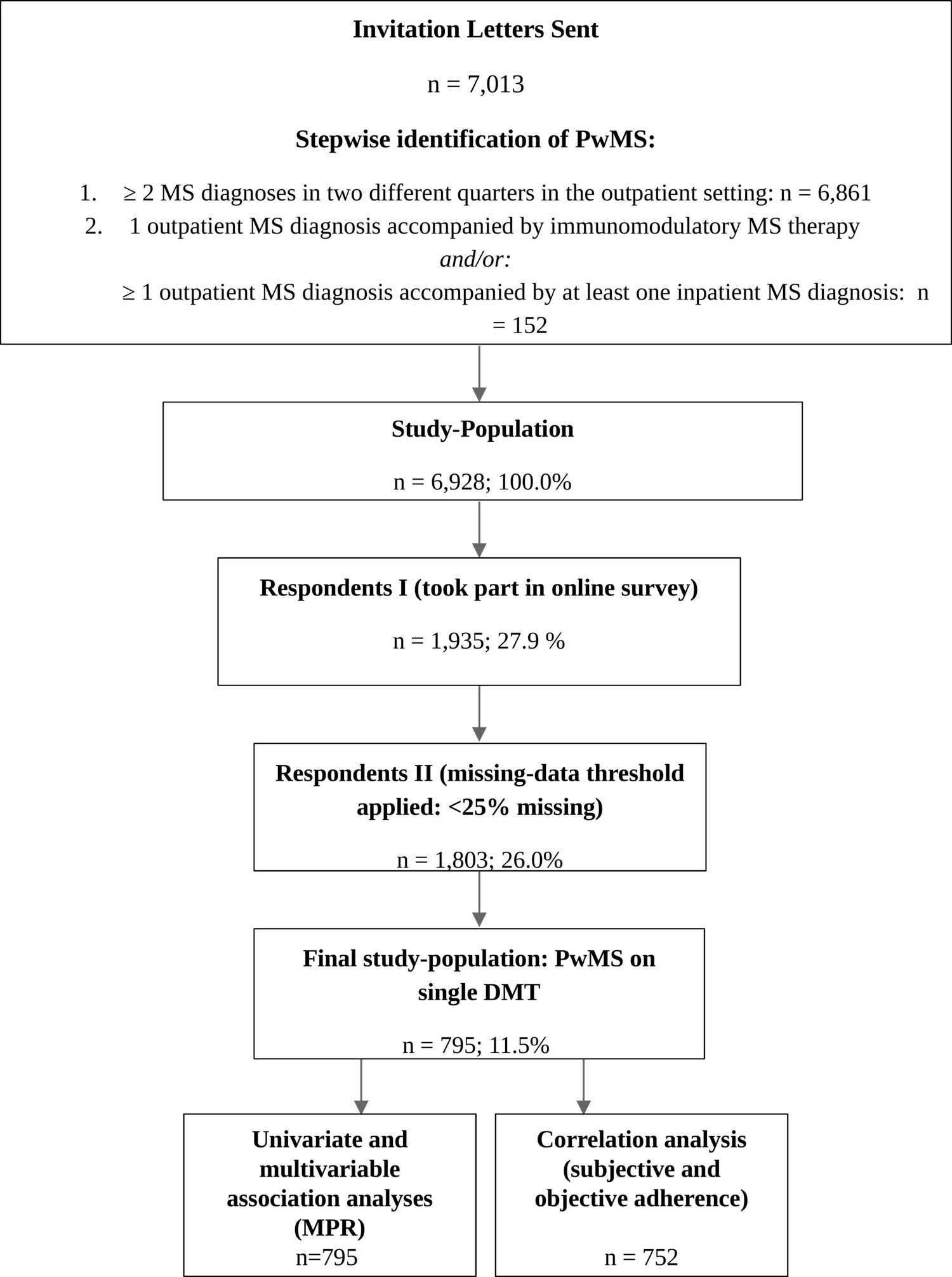
Participant flowchart. Flowchart of study population selection and analytical samples. Invitation letters were sent to 7,013 persons with multiple sclerosis (PwMS). After application of the case definition, 6,928 individuals were eligible, of whom 1,960 (28.3%) participated in the online survey. Respondents were classified as full responders (*n* = 679), partial responders (*n* = 1,256), and non-responders who opened but did not complete the survey (*n* = 25). Following stepwise exclusions based on eligibility and data completeness, 795 participants were included in the objective adherence analysis (medication possession ratio, MPR). For the correlation analysis between subjective and objective adherence, 752 participants with available visual analogue scale (VAS) data were included. MS = multiple sclerosis; PwMS = people with multiple sclerosis; DMT = disease-modifying therapy; MPR = medication possession ratio; VAS = visual analogue scale

Claims entries combining multiple routes or active substances within the observation year were excluded, as DMTs are prescribed as monotherapy and such records most plausibly reflect therapy switches, precluding substance- and route-specific MPR estimation. The analysis was therefore restricted to prevalent users with a single, stable, self-administered DMT regimen, capturing adherence to ongoing therapy rather than treatment transitions. Participants without a valid visual analogue scale response were included in MPR analyses but excluded from subjective–objective correlation analyses. Figure 1 summarises the selection process and stepwise exclusions. Full methodological details have been described previously (Fricke et al. 2024).

### Outcome measures

The primary outcome was objective adherence, measured as MPR. It was calculated as the ratio of the total days’ supply dispensed during the observation period to the number of days in the observation period and capped at 100% (Hess et al. 2006; Sperber et al. 2017). The total days’ supply was derived from route-specific defined daily doses based on the 2021 German ATC–DDD classification (Fricke et al. 2021) (Online Resource 1, Supplementary Table 2). Subjective adherence was assessed using a visual analogue scale and captures intake as perceived by PwMS (Online Resource 1, Supplementary Fig. 1).

### Statistical analysis

Statistical significance was defined as *p* < 0.05. Bivariate differences in objective adherence were assessed using Kruskal–Wallis tests (ε^2^ as effect size), with Dunn–Bonferroni post hoc tests applied where appropriate. Subgroups with *n* < 5 were retained for descriptive purposes but excluded from inferential analyses due to insufficient variance.

To quantify the relative contribution of clinical, demographic, and pharmacological domains, a permutational multivariate analysis of variance (PERMANOVA) with 100 permutations was used. PERMANOVA was chosen because the MPR distribution is bounded, non-normal, and heteroscedastic, and the method allows variance partitioning across categorical domains without imposing distributional assumptions (Anderson 2001; Anderson et al. 2017). A bootstrapped permutational model with 100 resamples served as a sensitivity analysis. Homogeneity of dispersion was assessed using Kruskal–Wallis comparisons of group variances. Missing survey data were imputed using non-parametric methods described elsewhere (Fricke et al. 2024). Descriptive analyses were conducted on the non-imputed dataset, whereas inferential analyses were based on the imputed dataset. Subjective–objective correlation was assessed by Spearman’s rank correlation (Online Resource 1, Supplementary Fig. 2). All analyses were conducted in RStudio (R version 4.3.2) and Python (version 3.11.3).

## Results

### Study population

The final cohort comprised 795 PwMS receiving non-intravenous DMTs. Women represented 71.1% of participants. Most PwMS were aged 36–65 years (80.2%), while 16.8% were 18–35 years and 3.0% were ≥ 66 years. A stable relationship was reported by 80.8% (Table 1).

**Table 1:** Descriptive characteristics of the study population (*N* = 795)

1. Sociodemographic Characteristics (*N* = 795)			
		*n*	%
Age ^†^	18-35 years	133	16.73
	36-50 years	327	41.13
	51-65 years	311	39.12
	66 years and older	24	3.02
	Missing values	0	0
Sex ^†^	Male	230	28.93
	Female	565	71.07
	Missing values	0	0
Employment Status ^‡^	Employed	503	63.27
	Not employed	287	36.10
	Missing values	5	0.63
Equivalised Household Net Income ^‡^	No risk of poverty	422	53.08
	Risk of poverty	309	38.87
	Missing values	64	8.05
Educational Status ^‡^	High	69	8.68
	Moderate	472	59.37
	Low	191	24.03
	Other	49	6.16
	Missing values	14	1.76
Marital Status ^‡^	Serious relationship	642	80.75
	Non-serious relationship	148	18.62
	Missing values	5	0.63
Region of Residence ^†^	Urban	151	18.99
	Suburban	268	33.71
	Rural	376	47.3
	Missing values	0	0
Living Situation ^‡^	With family or spouse	670	84.28
	Alone	107	13.46
	Other	16	2.01
	Missing values	2	0.25
Children in Household ^‡^	Two or more	88	11.07
	One	94	11.82
	None	613	77.11
	Missing values	0	0
2. Healthcare Access, Comorbidity, and Treatment-Related Factors (*N* = 795)			
Type of Therapy ^†^	IM	54	6.79
	SC	321	40.38
	O	420	52.83
	Missing values	0	0
Active Ingredients ^†^	S1P Receptor Modulators	127	15.97
	Interferons	206	25.91
	Glatiramer acetate	169	21.26
	Dimethyl fumarate	165	20.75
	Teriflunomide	119	14.97
	Azathioprine	6	0.75
	Cladribine	3	0.38
	Missing values	0	0
Comorbidity Index ^‡^	None	453	56.98
	1-2	247	31.07
	3-4	72	9.06
	5 or more	23	2.89
	Missing values	0	0
Main Medical Service Provider ^‡^	Specialised MS centre	52	6.54
	Outpatient neurologist	725	91.19
	General practitioner	16	2.01
	None	1	0.13
	Missing values	1	0.13
Nursing Care Needs ^‡^	Significant impairment	82	10.31
	No or little impairment	706	88.81
	Missing values	7	0.88
3. Clinical Course and Psychological Factors (*N* = 795)			
MS Disease Course ^‡^	Relapsing	510	64.15
	Non-relapsing	129	16.23
	Unknown	149	18.74
	Missing values	7	0.88
Duration of MS ^‡^	Up to 5 years	233	29.31
	6-10 years	175	22.01
	11-15 years	138	17.36
	16-25 years	186	23.4
	Over 25 years	46	5.79
	Unknown	13	1.64
	Missing values	4	0.5
Disease Progression ^‡^	More	398	50.06
	Same	321	40.38
	Less	42	5.28
	Missing values	34	4.28
Time to Diagnosis ^‡^	More than 6 years	120	15.09
	2-6 years	181	22.77
	Up to 1 year	422	53.08
	After Diagnosis	16	2.01
	Unknown	52	6.54
	Missing values	4	0.5
General Self-Efficacy ^‡^	High self-efficacious	376	47.3
	Low self-efficacious	381	47.92
	Missing values	38	4.78
Control Preferences ^‡^	Collaborative	409	51.45
	Active	299	37.61
	Passive	85	10.69
	Missing values	2	0.25

Table 1 shows clinical, demographic, and pharmacological characteristics of persons with multiple sclerosis included in the analysis. Variables were derived from health insurance claims or the patient survey, as indicated. Values are presented as absolute numbers and percentages; missing data are reported separately. *MS* multiple sclerosis, *IM* intramuscular, *SC* subcutaneous, *O* oral, *S1P* sphingosine-1-phosphate receptor modulators. ^†^taken from claims data, ^‡^taken from questionnaire data

Employment was reported by 63.3% of participants, and 38.9% indicated a risk of poverty. Educational attainment was predominantly moderate (59.4%), with 24.0% reporting low and 8.7% high educational levels. Participants primarily lived in rural (47.3%) or suburban (33.7%) areas; 19.0% lived in urban settings (Table 1).

Self-efficacy was evenly distributed (47.3% high; 47.9% low). A collaborative decision-making preference (i.e. collaborative treatment decisions between patient and clinician) was reported by 51.5% of participants (Table 1).

Relapsing forms of MS were present in 64.2% of the cohort. More than half (53.1%) had received their diagnosis within one year of symptom onset, and 50.1% reported disease progression in recent years (Table 1). Nursing care dependency was rare (10.3%). Outpatient neurologists were the main treatment providers for MS (91.2%), followed by MS specialist centres (6.5%) and general practitioners (2.0%) (Table 1).

Oral DMTs were most frequently prescribed (52.8%), followed by subcutaneous (40.4%) and intramuscular (6.8%) formulations. Interferons (25.9%) and glatiramer acetate (21.3%) were the most common active substances (Table 1).

Objective adherence, assessed via the MPR, averaged 88.4% (SD 18.0). Subjective adherence, assessed by visual analogue scale, averaged 95.9% (SD 11.6; median 100%), with 77.2% of participants reporting full adherence. The distribution of objective and subjective adherence is shown in Online Resource 1, Supplementary Fig. 1.

### Bivariate analysis of adherence predictors

Therapy-related variables showed the strongest associations with objective adherence. The composite therapy variable, combining type of therapy and active ingredient, explained approximately 12% of the variance in MPR (ε^2^ = 0.119, *p* < 0.001). Type of therapy alone accounted for approximately 10% (ε^2^ = 0.102), and active ingredient for approximately 11% (ε^2^ = 0.107; both *p* < 0.001). Full statistical results are presented in Table 2.

**Table 2:** Predictors of objective adherence

Variable	*N* (%)	Median MPR [IQR]	MPR ≥80%, n (%)	VAS = 100%, *n* (%)	Kruskal-Wallis H		Effect size	
					H test statistic	df	*p*-value	Epsilon Squared
Active Ingredients					92.13	6	<0.001***	0.107
Interferons	206 (25.91)	96.93 [75.71-100.00]	72.8% (150/206)	82.0% (159/194)				
IFN SC	152 (19.12)	95.59 [55.71–100.00]	66.4% (101/152)	82.6% (119/144)				
IFN IM	54 (6.79)	99.25 [92.30–100.00]	90.7% (49/54)	80.0% (40/50)				
Glatiramer acetate	169 (21.26)	86.54 [79.62-93.69]	72.8% (123/169)	80.0% (128/160)				
Dimethyl fumarate	165 (20.75)	99.32 [90.00-100.00]	89.7% (148/165)	73.5% (114/155)				
S1P Receptor Modulators	127 (15.97)	100.00 [89.10-100.00]	87.4% (111/127)	84.9% (101/119)				
Teriflunomide	119 (14.97)	100.00 [93.45-100.00]	93.3% (111/119)	87.0% (100/115)				
Azathioprine	6 (0.75)	68.57 [42.06-94.26]	50.0% (3/6)	83.3% (5/6)				
Cladribine	3 (0.38)	84.03 [77.88-92.02]	66.7% (2/3)	66.7% (2/3)				
Type of Therapy					85.04	2	<0.001***	0.102
O	420 (52.83)	100.00 [90.32-100.00]	89.3% (375/420)	80.9% (322/398)				
SC	321 (40.38)	88.95 [76.15-99.29]	69.8% (224/321)	81.2% (247/304)				
IM	54 (6.79)	99.25 [92.30-100.00]	90.7% (49/54)	80.0% (40/50)				
Therapy					102.75	7	<0.001***	0.119
IFN SC	152 (19.12)	95.59 [55.71–100.00]	66.4% (101/152)	82.6% (119/144)				
IFN IM	54 (6.79)	99.25 [92.30–100.00]	90.7% (49/54)	80.0% (40/50)				
SC / Glatiramer acetate	169 (21.26)	86.54 [79.62–93.69]	72.8% (123/169)	80.0% (128/160)				
O / Dimethyl fumarate	165 (20.75)	99.32 [90.00–100.00]	89.7% (148/165)	73.5% (114/155)				
O / S1P Receptor Modulators	127 (15.97)	100.00 [89.10–100.00]	87.4% (111/127)	84.9% (101/119)				
O / Teriflunomide	119 (14.97)	100.00 [93.45–100.00]	93.3% (111/119)	87.0% (100/115)				
O / Azathioprine	6 (0.75)	68.57 [42.06–94.26]	50.0% (3/6)	83.3% (5/6)				
O / Cladribine	3 (0.38)	84.03 [77.88–92.02]	66.7% (2/3)	66.7% (2/3)				
Age (years)					2.92	3	0.403	0
18-35	133 (16.73)	93.51 [84.21-100.00]	85.0% (113/133)	74.8% (92/123)				
36-50	327 (41.13)	97.18 [84.21-100.00]	81.0% (265/327)	78.4% (243/310)				
51-65	311 (39.12)	96.44 [84.81-100.00]	79.7% (248/311)	85.9% (256/298)				
66 and older	24 (3.02)	99.32 [91.08-100.00]	91.7% (22/24)	85.7% (18/21)				
Sex					0.07	1	0.792	0
Male	230 (28.93)	97.53 [85.96-100.00]	83.9%(193/230)	80.4% (172/214)				
Female	565 (71.07)	96.30 [84.00-100.00]	80.5% (455/565)	81.2% (437/538)				
Employment Status					0.026	1	0.880	0
Employed	504 (63.4)	96.83 [84.65-100.00]	82.1% (414/504)	79.2% (381/481)				
Not employed	291 (36.6)	96.15 [84.35-100.00]	80.4% (234/291)	84.1% (228/271)				
Household Income					0.02	1	0.896	0
No risk of poverty	467 (58.74)	96.53 [84.85-100.00]	81.6% (381/467)	81.2% (363/447)				
Risk of poverty	328 (41.26)	96.84 [84.35-100.00]	81.4% (267/328)	80.7% (246/305)				
Educational Status					1.59	3	0.662	0
Low	203 (25.53)	96.45 [84.19-100.00]	82.3% (167/203)	85.3% (163/191)				
Moderate	473 (59.5)	96.55 [84.75-100.00]	81.6% (386/473)	80.0% (361/451)				
High	70 (8.81)	96.29 [82.09-100.00]	78.6% (55/70)	73.1% (49/67)				
Other	49 (6.16)	97.03 [87.07-100.00]	81.6%(40/49)	83.7% (36/43)				
Marital Status					0.02	1	0.882	0
Serious relationship	647 (81.38)	96.55 [85.06-100.00]	81.6% (528/647)	81.3% (497/611)				
Non-serious relationship	148 (18.62)	96.68 [83.79-100.00]	81.1% (120/148)	79.4% (112/141)				
Region of Residence					3.06	2	0.217	0
Rural	376 (47.3)	96.40 [84.82-100.00]	83.0% (312/376)	81.6% (292/358)				
Suburban	268 (33.71)	95.88 [82.84-100.00]	78.4% (210/268)	80.2% (203/253)				
Urban	151 (18.99)	98.64 [87.10-100.00]	83.4% (126/151)	80.9% (114/141)				
Living Situation					1.08	2	0.582	0
With family or spouse	672 (84.53)	96.35 [84.51-100.00]	81.2% (546/672)	80.8% (513/635)				
Alone	107 (13.46)	98.00 [84.81-100.00]	84.1% (90/107)	81.2% (82/101)				
Other	16 (2.01)	100.00 [87.66-100.00]	75.0% (12/16)	87.5% (14/16)				
Children in Household					2.41	2	0.299	0
Two or more	88 (11.07)	93.47 [81.53-100.00]	78.4% (69/88)	70.9% (61/86)				
One	102 (12.83)	95.18 [84.05-100.00]	80.4% (82/102)	69.5% (66/95)				
None	605 (76.1)	97.23 [85.44-100.00]	82.1%(497/605)	84.4% (482/571)				
Comorbidity Index					7.78	3	0.198	0
5 or more	23 (2.89)	98.39 [88.84-100.00]	95.7% (22/23)	73.7% (14/19)				
3-4	72 (9.06)	98.64 [86.59-100.00]	81.9% (59/72)	92.6% (63/68)				
1-2	247 (31.07)	94.49 [82.52-100.00]	79.8% (197/247)	78.6% (187/238)				
None	453 (56.98)	96.84 [84.85-100.00]	81.7% (370/453)	80.8% (345/427)				
Main Medical Service Provider					1.34	4	0.855	0
Outpatient neurologist	725 (91.19)	96.55 [84.52-100.00]	81.4% (590/725)	81.1% (555/684)				
Specialised MS centre	52 (6.54)	96.30 [85.72-100.00]	82.7% (43/52)	84.0% (42/50)				
General practitioner	16 (2.01)	95.26 [83.98-100.00]	81.2% (13/16)	68.8% (11/16)				
Other	1 (0.13)	100.00 [100.00-100.00]	100.0% (1/1)	100.0% (1/1)				
None	1 (0.13)	97.84 [97.84-97.84]	100.0% (1/1)	0.0% (0/1)				
Nursing Care Needs					0.63	2	0.729	0
No or little impairment	706 (88.81)	96.49 [84.56-100.00]	81.6% (576/706)	80.4% (539/670)				
Significant impairment	82 (10.31)	98.10 [84.49-100.00]	80.5% (66/82)	85.3% (64/75)				
None	7 (0.88)	97.83 [92.67-100.00]	85.7% (6/7)	85.7% (6/7)				
Course of MS					0.59	2	0.745	0
Relapsing	516 (64.91)	96.74 [85.08-100.00]	81.2% (419/516)	81.3% (401/493)				
Non-relapsing	130 (16.35)	96.91 [85.40-100.00]	82.3% (107/130)	80.5% (95/118)				
Unknown	149 (18.74)	96.39 [82.80-100.00]	81.9% (122/149)	80.1% (113/141)				
Duration of MS					1.95	5	0.856	0
Up to 5 years	233 (29.31)	95.43 [85.73-100.00]	84.1% (196/233)	78.4% (174/222)				
6-10 years	175 (22.01)	96.55 [84.02-100.00]	80.0% (140/175)	79.0% (128/162)				
11-15 years	139 (17.48)	96.96 [84.92-100.00]	82.7% (115/139)	83.0% (112/135)				
16-25 years	186 (23.4)	97.18 [82.85-100.00]	76.9% (143/186)	84.0% (147/175)				
Over 25 years	49 (6.16)	98.00 [86.81-100.00]	85.7% (42/49)	83.0% (39/47)				
Unknown	13 (1.64)	92.31 [83.36-97.66]	92.3% (12/13)	81.8% (9/11)				
Disease Progression					2.12	2	0.346	0
More	399 (50.19)	96.41 [83.37-100.00]	80.5% (321/399)	80.2% (299/373)				
Same	354 (44.53)	96.63 [85.51-100.00]	82.8% (293/354)	81.2% (276/340)				
Less	42 (5.28)	100.00 [88.25-100.00]	81.0% (34/42)	87.2% (34/39)				
Time to Diagnosis					1.90	4	0.754	0
Up to 1 year	422 (53.08)	97.18 [84.89-100.00]	82.5% (348/422)	80.2% (321/400)				
2-6 years	183 (23.02)	96.79 [84.77-100.00]	81.4% (149/183)	84.1% (148/176)				
More than 6 years	121 (15.22)	94.49 [85.71–100.00]	78.5% (95/121)	77.0% (87/113)				
Unknown	52 (6.54)	96.81 [83.29–100.00]	84.6% (44/52)	84.8% (39/46)				
After Diagnosis	17 (2.14)	95.92 [79.85–100.00]	70.6% (12/17)	82.4% (14/17)				
Control Preferences (CPS)					0.97	3	0.807	0
Collaborative	410 (51.57)	96.15 [85.71–100.00]	82.7% (339/410)	83.8% (327/390)				
Active	299 (37.61)	97.03 [83.23–100.00]	80.6% (241/299)	76.4% (217/284)				
Passive	85 (10.69)	98.40 [84.17–100.00]	78.8% (67/85)	84.4% (65/77)				
None	1 (0.13)	97.84 [97.84-97.84]	100.0%(1/1)	0.0% (0/1)				
General Self-Efficacy (GSE)					0.035	1	0.851	0
Low self-efficacious	400 (50.31)	96.83 [84.03–100.00]	79.5% (318/400)	78.8% (293/372)				
High self-efficacious	395 (49.69)	96.39 [84.99–100.00]	83.5% (330/395)	83.2% (316/380)				

Table 2 summarises univariate associations between patient characteristics and adherence. Objective adherence was measured as the Medication Possession Ratio (MPR), calculated from pharmacy claims data. Subjective adherence was assessed using a visual analogue scale (VAS; Online Resource 1, Supplementary Fig. 1). Kruskal–Wallis tests indicated that only pharmacological variables were significantly associated with variation in MPR, showing the largest effect sizes. Clinical and demographic variables showed no significant associations. Counts and percentages are based on the imputed dataset (*N* = 795). Effect sizes are reported as epsilon squared (ε²). *p < 0.05, **p < 0.01, ***p < 0.001. *CPS* control preferences scale, *df* degrees of freedom, *GSE* general self-efficacy, *IFN* interferon, *IM* intramuscular, *IQR* interquartile range, *MPR* medication possession ratio, *MS* multiple sclerosis, *N* number, *O* oral, *S1P* sphingosine-1-phosphate receptor modulators, *SC* subcutaneous, *VAS* visual analogue scale, *ε²* epsilon squared

Demographic characteristics, including age and sex, showed no association with adherence (all *p* > 0.05). Socioeconomic characteristics, including employment status, household income, and educational attainment, also contributed no measurable explanatory value (Table 2).

### Pairwise comparisons of therapy modalities

#### Route of administration

Adherence was comparable between oral agents and intramuscular interferon beta-1a, the only intramuscular DMT represented, and was higher than for subcutaneous therapies (oral vs subcutaneous and intramuscular vs subcutaneous, both *p* < 0.001; oral vs intramuscular, *p* = 0.90) (Table 3).

**Table 3:** Pairwise comparisons of therapy modalities

Therapy A	Therapy B	*p*-value	Interpretation
IM	O	*0.9016*	n.s.
IM	SC	*<0.001*	IM > SC
O	SC	*<0.001*	O > SC

#### Active substances

Adherence differed substantially across agents. Glatiramer acetate showed the lowest levels, whereas teriflunomide, sphingosine-1-phosphate (S1P) receptor modulators, and dimethyl fumarate showed the highest (all *p* < 0.001 vs glatiramer acetate); interferons also exceeded glatiramer acetate (*p* < 0.001). Interferons showed lower adherence than dimethyl fumarate (*p* < 0.05), S1P receptor modulators (*p* = 0.01), and teriflunomide (*p* < 0.001) (Table 4).

**Table 4:** Pairwise comparisons of active ingredients

Comparison	*p*-value	Interpretation
Glatiramer acetate vs. Teriflunomide	<0.001	Teriflunomide > Glatiramer acetate
Glatiramer acetate vs. S1P Modulators	<0.001	S1P Modulators > Glatiramer acetate
Glatiramer acetate vs. Dimethyl fumarate	<0.001	Dimethyl fumarate > Glatiramer acetate
Glatiramer acetate vs. Interferons	0.00025	Interferons > Glatiramer acetate
Dimethyl fumarate vs. Interferons	0.0346	Dimethyl fumarate > Interferons

Azathioprine and cladribine were not compared because of limited within-group variability. Stratification of interferons by route showed higher adherence for intramuscular than for subcutaneous formulations (MPR ≥ 0.8: 90.7% vs 66.4%; *p* = 0.002) (Table 2). The aggregate interferon estimate therefore reflects the distribution of these subgroups. This within-substance contrast mirrors the route-related gradient observed across therapies.

### Multivariate analysis of adherence determinants

#### Therapy-related factors

Permutation-based multivariable models confirmed that therapy characteristics were the only meaningful predictors of adherence. Type of therapy and active ingredient explained the largest shares of variance (both *p* < 0.001), consistent with the bivariate results (Table 5).

**Table 5:** Multivariate determinants of objective adherence

Variable	pseudo-F	*p*-value
Type of therapy	37.595	<0.001
Active ingredients	11.571	<0.001
Employment status	0.407	0.521
Marital status	0.384	0.533
General Self Efficacy (GSE)	0.999	0.322
Household income	0.228	0.625
Children in household	0.017	0.98
Living situation	0.881	0.414
Time from symptom onset to diagnosis	0.207	0.942
Course of multiple sclerosis	0.062	0.938
Disease progression	0.489	0.595
Relapses during the past 24 months	1.431	0.238
Main medical service provider	0.262	0.844
Control preferences (CPS)	0.113	0.957
Educational status	0.721	0.536
Region of residence	0.534	0.563
Level of nursing care	0.232	0.801
Sex	3.837	0.058
Age	0.695	0.55
Duration of multiple sclerosis	1.086	0.352
Comorbidity index	0.746	0.522

#### Other domains

Demographic, socioeconomic, clinical and psychological variables, including age, sex, employment, income, disease course, diagnostic delay, comorbidity burden, recent disease progression, self-efficacy and decision-making preference, were not associated with objective adherence (all *p* > 0.05) (Table 5).

#### Supplementary analyses

Bootstrapped permutational models produced comparable results (Online Resource 1, Supplementary Table 3 and Supplementary Table 4).

#### Correlation between subjective and objective adherence

Subjective adherence showed only a weak association with MPR (Spearman’s ρ = 0.10, *p* = 0.005). The distribution of self-reported values was strongly right-skewed, with a pronounced ceiling effect (median 100%; 95.4% reporting ≥ 80%), which constrains the observable correlation range. Although statistically significant, the correlation indicates limited correspondence between self-reported adherence and dispensing-based measures (Online Resource 1, Supplementary Fig. 2).

## Discussion

This study demonstrates that treatment-specific characteristics are the dominant determinants, within the examined domains, of adherence to DMTs in PwMS. Oral and intramuscular therapies, notably teriflunomide, S1P receptor modulators, and dimethyl fumarate, achieved the highest adherence, whereas glatiramer acetate the lowest. Clinical and demographic variables, including socioeconomic indicators, showed no measurable association. The proportion of variance attributable to treatment factors exceeded earlier reports (Steiner and Prochazka 1997; Washington and Langdon 2022), suggesting that, in routine care with universal healthcare access, adherence is shaped primarily by pharmacological properties and the conditions under which therapies are delivered.

The observation period coincided with the first year of the SARS-CoV-2 pandemic in Germany, during which care was delivered under altered conditions, including reduced in-person contact, partial substitution by telemedicine (Reitzle et al. 2021; Platen et al. 2022), and a shift towards self-administered DMTs (Portaccio et al. 2022; Konitsioti et al. 2025). MPR-based estimates may have been influenced by changes in dispensing behaviour, including extended prescription intervals and stockpiling (Olmastroni et al. 2023). However, longitudinal pharmacy dispensing data from Germany indicate stable DMT prescription volumes between 2019 and 2021, with stockpiling limited to the initial pandemic phase and not observed during the present observation period (Orschiedt et al. 2023).

Adherence patterns were not explained by route category alone. Intramuscular interferon beta-1a showed adherence comparable to that of oral therapies and consistently higher than that of subcutaneous therapies. The within-interferon comparison provides the strongest internal evidence: adherence was higher for intramuscular than for subcutaneous interferon despite the same active substance, indicating that route-associated regimen characteristics, rather than pharmacological differences alone, drive the observed gradient. Similar patterns have been reported in German (Hansen et al. 2015) and US cohorts (Halpern et al. 2011), supporting a stable, non-cohort-specific effect.

Several non-exclusive regimen characteristics may contribute, including the lower dosing frequency for intramuscular interferon beta-1a, differences in injection-site tolerability and immunogenicity (Beer et al. 2011; Cohan et al. 2021), and cumulative local adverse effects associated with long-term subcutaneous therapies such as glatiramer acetate. These mechanisms align with patient-preference data showing that reduced dosing frequency is valued similarly to gains in efficacy and is associated with higher satisfaction and adherence (Poulos et al. 2018; Cutter et al. 2019), although they were not directly measured here. Structured support for subcutaneous therapies in Germany, including nurse-led training, follow-up, and manufacturer-supported programmes (Schwab et al. 2024), is not uniformly covered. Adherence to oral DMTs remains heterogeneous (Nicholas et al. 2020), indicating that regimen characteristics beyond route influence adherence.

The prominence of therapy-related predictors underscores the central role of treatment attributes and delivery context in shaping adherence in routine MS care. Although multiple demographic, socioeconomic, and clinical covariates were considered, residual confounding remains likely, as prescribing may reflect unobserved decision processes such as escalation strategies or clinician preference. While patient decision-making preference was included as a behavioural indicator, it reflects a general orientation towards treatment involvement rather than specific decision-making processes or preference-concordant treatment selection. This limitation indicates that, although behavioural influences remain relevant, they may not have been captured at a level sufficient to account for variation in objective adherence.

Interpreting adherence requires caution. Common thresholds such as 80% have not been validated for MS and may not represent clinically meaningful exposure (Baumgartner et al. 2018). The weak correlation between subjective and objective adherence aligns with prior research showing that self-report reflects perceptions of behaviour, whereas dispensing data measure medication availability rather than ingestion (Stirratt et al. 2015; Ihle et al. 2019). The marked ceiling effect in self-reported values, with more than three-quarters of participants reporting full adherence, is consistent with social desirability and recall bias and further attenuates concordance with pharmacy-based metrics. These measures therefore capture distinct constructs, supporting the use of multiple adherence assessments in research and clinical practice.

Taken together, these findings suggest that adherence interventions should prioritise treatment selection and delivery, aligning therapy characteristics with patient capability and available support structures. Demographic and socioeconomic factors showed no explanatory value and are therefore less promising targets. These findings apply to self-administered DMTs and cannot be generalised to intravenous or combination therapies. The persistence of lower adherence for subcutaneous therapies in pre-pandemic data (Hansen et al. 2015) argues against pandemic-related factors as the primary explanation. The weak correspondence between subjective and objective adherence supports the integration of objective monitoring (Stirratt et al. 2015). Future studies should incorporate patient preferences, cognitive and functional measures, and system-level determinants. Longitudinal designs including high-efficacy and clinic-administered therapies are needed to clarify causal pathways and inform personalised adherence strategies.

## Limitations

The cross-sectional design does not permit causal inference. Although permutation-based multivariable analyses are well suited to non-normally distributed outcomes, they do not provide effect estimates on an additive scale.

Claims data capture dispensing rather than ingestion and cannot account for dose adjustments or distinguish intentional from unintentional non-adherence (Hempenius et al. 2021). They also lack clinical detail, including MRI activity, which may influence adherence but was unavailable. Coding misclassification is possible, although unlikely to be systematically related to adherence. The observation period overlapped with the first year of the SARS-CoV-2 pandemic in Germany, during which healthcare delivery and treatment selection were systematically modified (Platen et al. 2022; Reitzle et al. 2021; Portaccio et al. 2022). German pharmacy data nevertheless indicate that overall DMT dispensing remained stable across 2019–2021, with no systematic disruption observed during the present observation window (Orschiedt et al. 2023). Voluntary survey participation introduces selection bias, and subjective measures remain vulnerable to recall and social desirability effects. Missing survey data were imputed using validated non-parametric procedures; while imputation introduces uncertainty, sensitivity analyses yielded consistent findings.

The adherence metric limits generalisability. MPR is derived from dispensing records and depends on patient-initiated refills, rendering intravenous therapies non-evaluable and limiting the analysis to self-administered DMTs.

The intramuscular group was small (*n* = 54; 6.8%), and although the oral–intramuscular difference was not statistically significant, this comparison was estimated less precisely than those involving larger groups. Route and active substance are structurally aligned across available DMTs: the intramuscular group comprises a single once-weekly formulation, whereas the subcutaneous group includes multiple agents with differing schedules and tolerability profiles. Cross-route comparisons therefore reflect combined regimen characteristics rather than isolated route effects, although the within-interferon comparison indicates that route remains relevant when active substance is held constant. MPR quantifies dispensing relative to a label-based reference and is therefore sensitive to differences in prescribed regimens, but it does not identify which regimen-specific features account for the observed effects.

## Conclusion

Within the spectrum of self-administered DMTs examined, adherence tracked characteristics of the prescribed regimen, particularly route of administration and active substance, rather than patient demographics or clinical profile. Oral agents and intramuscular interferon beta-1a showed similarly high adherence, with no significant difference between them, whereas subcutaneous therapies, and glatiramer acetate in particular, showed consistently lower adherence. This gradient has been reported in German and US claims-based cohorts (Hansen et al. 2015; Halpern et al. 2011) and is consistent across care settings and observation periods. An interferon-based comparison, in which intramuscular and subcutaneous formulations of the same active substance differed substantially in adherence, indicates that route of administration contributes to the observed gradient beyond active substance alone. The dispensing-based measure and the restriction to self-administered therapies allow a methodologically consistent assessment of MPR, but limit generalisability to the full spectrum of DMTs used in routine care. The weak correspondence between subjective and objective adherence underscores the need for objective monitoring; the measures capture complementary, rather than interchangeable, constructs.

These findings support prioritising treatment selection and delivery models that facilitate sustained adherence. This includes strengthening structured support for patients receiving subcutaneous therapies, aligning treatment with patient capability, and ensuring continuity of follow-up. At a policy level, investment in adherence-supportive infrastructure may be more effective than targeting demographic risk groups, which showed no predictive value in this cohort. Future longitudinal studies incorporating functional outcomes, patient preference data, and clinic-administered therapies are required to clarify causal pathways and inform adherence strategies responsive to both patient needs and treatment demands.

## Supplementary Information

Below is the link to the electronic supplementary material.

Supplementary file1 (DOCX 3289 KB)

## Data Availability

The datasets generated and/or analysed during the current study are not publicly available due to data protection regulations and contractual restrictions related to statutory health insurance claims data, but are available from the corresponding author on reasonable request, subject to approval by the data holders and applicable legal and ethical requirements.

## Declarations

### Use of digital tools

Digital tools were used during the preparation of this manuscript. In particular, an AI-based large language model was used to assist with language editing, structuring, and refinement of text passages. The conceptual development of the study, data collection, statistical analyses, interpretation of the results, and scientific responsibility for all content remain solely with the authors.
