# Supplementary material for: Clinical, demographic, and pharmacological predictors of medication adherence to disease-modifying therapies in multiple sclerosis: a multidimensional analysis of merged survey and claims data
Source: Naunyn Schmiedebergs Arch Pharmacol. 2026 May 26;399(11):17649–63. doi: 10.1007/s00210-026-05448-w (PMC13503424; doi:10.1007/s00210-026-05448-w)
Supplement: Supplementary file 1 [file 210_2026_5448_MOESM1_ESM.docx]

**SUPPLEMENTAL DIGITAL CONTENT**

**Clinical, Demographic, and Pharmacological Predictors of Medication Adherence to**

**Disease-Modifying Therapies in Multiple Sclerosis:**

**A Multidimensional Analysis of**

**Merged Survey and Claims Data.**

Ahkash Thavarajasingam¹

Lara Marleen Fricke¹,²

Christian Krauth¹,²

¹ Hannover Medical School, Institute for Epidemiology, Social Medicine and Health Systems Research,
Carl-Neuberg-Straße 1, 30625 Hannover, Germany

² Center for Health Economics Research Hannover (CHERH),

Hannover, Germany

Corresponding author: Ahkash Thavarajasingam

Email address: thavarajasingam.ahkash@mh-hannover.de

**Table of Contents:**

[**Supplementary Table 1. Overview of variables, sources, coding, and operational definitions used in the analysis.	3**](#_Toc223883325)

[**Supplementary Table 2: ATC-based classification and definitions of disease-modifying therapies.	6**](#_Toc223883326)

[**Supplementary Table 3: Bootstrap validation of the PERMANOVA model for objective adherence	6**](#_Toc223883327)

[**Supplementary Figure 1. Distributions of objective and subjective medication adherence	11**](#_Toc223883328)

[**Supplementary Figure 2. Illustration of Spearman’s rank correlation.	12**](#_Toc223883329)

**Supplementary Table 1. Overview of variables, sources, coding, and operational definitions used in the analysis.**

| Variable | Source | Original variable / raw format | Final coding in analysis | Operational definition |
| --- | --- | --- | --- | --- |
| Age | Claims data | Age category from insurance record | 18–35; 36–50; 51–65; ≥66 | Insurance-provided age categories regrouped to ensure sufficient group sizes for comparative analyses. |
| Sex | Claims data | Sex recorded from insurance record | Male / Female | As reported in insurance records. |
| Employment status | Questionnaire | Employment status items | Employed / Not employed | Dichotomised from self-reported work status. |
| Equivalised household net income (poverty risk) | Questionnaire | Household income group and equivalence weighting | Risk of poverty / No risk of poverty | Equivalised net income calculated following Robert Koch Institute (RKI) methodology; cut-off at €1,250.75 (60% of the national median equivalised income). |
| Educational status | Questionnaire | School and vocational qualification items | Low / Moderate / High / Other | Combined into an aggregate educational score following Robert Koch Institute (RKI) methodology and categorised into three socioeconomic status (SES) levels, with an additional Other category for non-mappable qualifications. |
| Marital status | Questionnaire | Partnership/relationship item | Serious relationship / Non-serious relationship | Self-reported relationship status, dichotomised. |
| Region of residence | Claims data | Regional type classification | Urban / Suburban / Rural | Based on the regional typology of the National Association of Statutory Health Insurance Physicians in Germany (types 1–5). |
| Living situation | Questionnaire | Household composition item | With family or spouse / Alone / Other | Self-reported living arrangement. |
| Children in household | Questionnaire | Number of children in household | None / One / Two or more | Self-reported household children count. |
| Type of therapy | Claims data | Route of administration (ATC-coded) | Oral / Subcutaneous / Intramuscular | Derived from the DMT administration route in prescription data (see Supplementary Table 2). |
| Active ingredients | Claims data | ATC-coded medication records | Interferons; Glatiramer acetate; Dimethyl fumarate; Teriflunomide; S1P receptor modulators; Azathioprine; Cladribine | Grouped according to pharmacological class based on the German Anatomical Therapeutic Chemical classification with defined daily doses, 2021 edition (WIdO, 2021); full mapping in Supplementary Table 2. |
| Comorbidity index (CCI) | Claims data | ICD-10 chronic-condition records | None / 1–2 / 3–4 / ≥5 | Charlson Comorbidity Index computed from claims ICD-10 codes. |
| Main medical service provider for multiple sclerosis (self-identified) | Questionnaire | Primary MS care provider item | Outpatient neurologist / Specialised MS centre / General practitioner / Other | Self-reported main MS care provider. |
| Level of nursing care | Questionnaire and claims data | German statutory long-term care classification (levels 0–5) | No or little impairment / Significant impairment | Statutory long-term care levels 2–5 coded as significant impairment; levels 0–1 coded as no or little impairment. |
| Course of multiple sclerosis | Questionnaire | MS course item | Relapsing / Non-relapsing / Unknown | Grouped in agreement with treating neurologists: relapsing course, non-relapsing course, or unknown. |
| Duration of multiple sclerosis | Questionnaire | Year of diagnosis | ≤5 / 6–10 / 11–15 / 16–25 / >25 years / Unknown | Time since first confirmed MS diagnosis, computed from self-reported year of diagnosis. |
| Recent disease impairment | Questionnaire | Change in MS-related impairment over the past 2 years | More / Same / Less | Self-reported change in severity of MS-related impairment. |
| Relapses during past 24 months | Questionnaire | Relapse count item | None / One or more / Unknown | Self-reported number of relapses in the past 24 months, collapsed into three categories for analysis. |
| Time from symptoms onset to diagnosis | Questionnaire | Years from first symptoms to diagnosis | ≤1 year / 2–6 years / >6 years / After diagnosis / Unknown | Diagnostic delay categories derived from patient report. |
| Hospital admissions | Claims data | Count of inpatient MS-coded admissions during the observation period | Continuous count (0–5) | Number of inpatient stays with an MS diagnosis recorded during the 12-month observation period (1 October 2020 – 30 September 2021). |
| General Self Efficacy (GSE) | Questionnaire | 10-item General Self-Efficacy Scale (sum score, range 10–40) | Low / High self-efficacious | Dichotomised by median split at a sum score of 30 (≤29 = low; ≥30 = high), following the operationalisation described by the scale authors (Schwarzer & Jerusalem, 1995). |
| Control preferences (CPS) | Questionnaire | Image-revised CPS (Solari et al., 2013) | Active / Collaborative / Passive | Five role-preference response options collapsed into three preference categories following the conventional grouping (Solari et al., 2013). |
| Objective Medication Adherence (MPR) | Claims data | Days’ supply dispensed within the observation period | Continuous 0–100% | Medication Possession Ratio; ratio of total days’ supply dispensed during the observation period to the number of days observed, capped at 100% (Hess et al., 2006; Sperber et al., 2017). Days’ supply derived from route-specific defined daily doses per the 2021 German ATC–DDD classification (WIdO, 2021). |
| Subjective Medication Adherence (VAS) | Questionnaire | Visual analogue scale (0–100%) | Continuous 0–100% | Self-reported adherence on a 100-point visual analogue scale; captures intake as perceived by the participant. |

Supplementary Table 1 provides an overview of all variables used in the analyses, including their data sources, raw formats, coding schemes, and operational definitions.

**Supplementary Table 2: ATC-based classification and definitions of disease-modifying therapies.**

| Drug class | Active substance | ATC code | Route of administration | Pharmacological group (according to WHO/ATC) | Example trade names (DE) |
| --- | --- | --- | --- | --- | --- |
| S1P receptor modulators | Fingolimod | L04AA27 | Oral | Sphingosine-1-phosphate receptor modulator | Gilenya |
|  | Siponimod | L04AA42 | Oral | S1P receptor modulator | Mayzent |
|  | Ozanimod | L04AA38 | Oral | S1P receptor modulator | Zeposia |
| Interferons | Interferon beta-1a | L03AB07 | SC or IM | Cytokines, immunomodulators | Avonex, Rebif |
|  | Interferon beta-1b | L03AB08 | SC | Cytokines, immunomodulators | Betaferon, Extavia |
|  | Peginterferon beta-1a | L03AB13 | SC | Pegylated interferon | Plegridy |
| Glatiramer acetate | Glatiramer acetate | L03AX13 | SC | Synthetic polypeptide immunomodulator | Copaxone |
| Fumarates | Dimethyl fumarate | L04AX07 | Oral | Fumaric acid esters | Tecfidera |
| Pyrimidine synthesis inhibitor | Teriflunomide | L04AA31 | Oral | Dihydroorotate dehydrogenase inhibitor | Aubagio |
| Antimetabolites | Azathioprine | L04AX01 | Oral | Purine analogue, immunosuppressant | Imurek |
| Purine analogues | Cladribine | L04AA40 | Oral | Synthetic deoxyadenosine analogue | Mavenclad |

Supplementary Table 2 lists all disease-modifying therapies identified in the cohort, including ATC codes, administration routes, pharmacological classes, and representative trade names used for classification in the analysis.

**Supplementary Table 3: Bootstrap validation of the PERMANOVA model for objective adherence**

| Predictor | Bootstrap mean pseudo-F | 95% bootstrap CI |
| --- | --- | --- |
| Active ingredients | 12.25 | 6.94 – 20.14 |
| Type of therapy | 36.04 | 17.97 – 55.02 |
| Comorbidity index | 1.49 | 0.26 – 3.63 |
| Duration of multiple sclerosis | 1.98 | 0.48 – 4.84 |
| Time from symptom onset to diagnosis | 1.10 | 0.15 – 2.75 |
| Age | 1.42 | 0.15 – 3.06 |
| Region of residence | 1.59 | 0.01 – 5.51 |
| Educational status | 1.65 | 0.12 – 4.81 |
| Children in household | 0.77 | 0.02 – 2.66 |
| Disease progression | 1.72 | 0.04 – 6.81 |
| Main medical service provider | 0.80 | 0.10 – 2.28 |
| Living situation | 1.21 | 0.05 – 3.47 |
| Course of multiple sclerosis | 1.24 | 0.03 – 4.90 |
| Level of nursing care | 0.88 | 0.07 – 2.95 |
| Control preferences (CPS) | 1.08 | 0.09 – 3.54 |
| Relapses during the past 24 months | 2.49 | 0.12 – 7.08 |
| Sex | 4.37 | 0.07 – 11.89 |
| General self-efficacy (GSE) | 1.85 | 0.00 – 7.42 |
| Employment status | 1.53 | 0.00 – 6.70 |
| Marital status | 1.38 | 0.00 – 6.76 |
| Household income | 1.26 | 0.00 – 5.82 |

Supplementary Table 3 depicts Bootstrap validation based on repeated resampling of the PERMANOVA model. The distribution of pseudo-F statistics across bootstrap samples showed a comparable hierarchy of effects, with pharmacological treatment variables exhibiting the strongest contributions to variation in objective adherence.

Supplementary Table 4: Complete bootstrap validation of multivariate determinants of objective adherence.

| Predictor | Bootstrap Mean Pseudo-F | 95% CI [Lower, Upper] | Mean p-value |
| --- | --- | --- | --- |
| Therapy (Composite) | 95.84 | [92.62, 99.06] | < 0.001 |
| Active ingredients | 86.51 | [83.48, 89.53] | < 0.001 |
| Type of therapy | 76.90 | [73.66, 80.15] | < 0.001 |
| Comorbidity Index | 7.45 | [6.41, 8.49] | 0.198 |
| Duration of multiple sclerosis | 6.97 | [6.25, 7.69] | 0.330 |
| Region of residence | 5.72 | [4.88, 6.56] | 0.196 |
| Time from symptoms onset to diagnosis | 5.22 | [4.51, 5.93] | 0.401 |
| Age | 5.17 | [4.49, 5.85] | 0.294 |
| Educational status | 4.21 | [3.66, 4.76] | 0.360 |
| Children in household | 4.01 | [3.32, 4.70] | 0.298 |
| Recent disease impairment | 3.64 | [2.95, 4.34] | 0.364 |
| Living situation | 3.27 | [2.73, 3.81] | 0.351 |
| Control preferences (CPS) | 3.22 | [2.66, 3.77] | 0.357 |
| Course of multiple sclerosis | 2.70 | [2.25, 3.14] | 0.390 |
| Relapses during past 24 months | 2.51 | [1.97, 3.06] | 0.448 |
| Level of nursing care | 2.12 | [1.72, 2.53] | 0.427 |
| Main medical service provider | 2.09 | [1.77, 2.41] | 0.452 |
| Sex | 0.07 | [0.06, 0.07] | 0.058* |
| General Self-Efficacy (GSE) | 0.04 | [0.03, 0.04] | 0.322* |
| Employment status | 0.04 | [0.03, 0.04] | 0.521* |
| Marital status | 0.03 | [0.03, 0.04] | 0.533* |
| Household income | 0.03 | [0.03, 0.04] | 0.625* |

**Supplementary Table 3** Bootstrap validation (1,000 iterations) of the PERMANOVA model for objective adherence (MPR). Reported are mean pseudo-F values, 95% bootstrap confidence intervals, and mean p-values. Pharmacological variables (active ingredients, therapy type, composite therapy) showed the largest and most stable effects (consistently high pseudo-F, narrow CIs, p < 0.001), confirming their dominant contribution. Clinical and demographic variables exhibited minimal effects (pseudo-F near zero, CIs approaching zero, non-significant p-values). Effect size hierarchy closely matches the primary multivariable model (Table 5), supporting robustness. For Sex, GSE, employment status, marital status, and household income, bootstrap p-values were not estimable due to negligible contributions. Asterisked (*) values correspond to the primary model (Table 5).

**Supplementary Fig. 1 Distributions of objective and subjective medication adherence**

**Supplementary Fig. 1** Distribution of objective adherence (Medication Possession Ratio, MPR; left) and self-reported adherence (visual analogue scale, VAS; right) in ten-percentage bands. MPR demonstrates a broad, heterogeneous distribution across adherence levels, whereas VAS scores collapse at the upper limit, with 95% of patients reporting ≥90% and a median of 100%. This pronounced ceiling effect underscores the limited resolution and discriminative validity of self-reported adherence compared with objective pharmacy refill measures.

**Supplementary Fig. 2 Illustration of Spearman’s rank correlation.**

**a**
**Supplementary Fig. 2** Self-reported adherence shows a strong ceiling effect and only a very weak monotonic association with objective adherence (Spearman’s ρ = 0.10), with no linear relationship

References

Schwarzer R, Jerusalem M, Weinman J, et al (1995) Generalized Self-Efficacy Scale. Measures in Health Psychology: A User’s Portfolio Causal and control beliefs Windsor
